# Local Administration of GITR Agonistic Antibody Induces a Stronger Antitumor Immunity than Systemic Delivery

**DOI:** 10.1038/s41598-019-41724-x

**Published:** 2019-04-03

**Authors:** Kenta Narumi, Reina Miyakawa, Chihiro Shibasaki, Marina Henmi, Yukihiro Mizoguchi, Ryosuke Ueda, Hisayoshi Hashimoto, Nobuyoshi Hiraoka, Teruhiko Yoshida, Kazunori Aoki

**Affiliations:** 1 Department of Immune Medicine, National Cancer Center Research Institute, 5-1-1 Tsukiji, Chuo-ku, Tokyo 104-0045 Japan; 2 Department of Molecular Pathology, National Cancer Center Research Institute, 5-1-1 Tsukiji, Chuo-ku, Tokyo 104-0045 Japan; 3 Fundamental Innovative Oncology Core, National Cancer Center Research Institute, 5-1-1 Tsukiji, Chuo-ku, Tokyo 104-0045 Japan

## Abstract

An anti-glucocorticoid induced TNF receptor (GITR) agonistic antibody (Ab) induces an antitumor immunity with both stimulation of effector T cells and inhibition of regulatory T cell activity. To enhance GITR Ab-mediated tumor immunity, we focused on the intratumoral route, since a tumor-localized high concentration of Ab would confer activation of only tumor-infiltrating T cells. First, in a murine colon cancer model, we showed that the intratumoral delivery of Ab significantly increased the number of effector T cells infiltrated into tumors, and suppressed tumor growth more effectively than the intraperitoneal and intravenous injections did. Then, we found that the injection of Ab into the peritumoral area induced a systemic antitumor immunity at a similar level to the intratumoral injection. Therefore, we hypothesized that the transfer of locally administrated Ab into tumor-draining lymph nodes (TDLNs) plays an important role in inducing an effective immunity. In fact, intratumorally or peritumorally injected Ab was detected in TDLNs, and resection of Ab-injected TDLNs significantly reduced GITR Ab-mediated systemic tumor immunity. Intratumoral injection showed less number of auto-reactive T cells in the spleen than the intraperitoneal injection did. Intratumoral delivery of GITR Ab is a promising approach to induce an effective immunity compared to the systemic delivery.

## Introduction

The field of cancer immunotherapy is expanding rapidly with the success of an antagonistic antibody against anti-cytotoxic T lymphocyte antigen-4 (CTLA-4)^1,2^. Subsequent to CTLA-4, programmed cell death receptor-1 (PD-1)/programmed cell death receptor-1-ligand-1 (PD-L1) targeted therapies are showing promising results^3,4^. However, since approximately half of patients do not respond to the therapies even the combination regimen, the development of novel checkpoint inhibitors is desired for the recurrent or refractory patients. Recently, newer targets including select members of the tumor necrosis factor receptor (TNFR) family, including 4-1BB, OX40 and glucocorticoid-induced tumor necrosis factor receptor (GITR), are gathering attention^5^. These molecules are expressed on both effector T cells and regulatory T cells (Tregs), and agonistic antibodies to them have provided useful tools for research into these co-stimulatory pathways^6^.

GITR was originally discovered as a gene upregulated in dexamethasone-treated murine T cell hybridomas^7^. Although dexamethasone treatment played a role in the discovery of GITR, it was shown that glucocorticoid treatment is unnecessary to achieve the function^8^. Similar to 4-1BB and OX40, GITR is expressed at a low basal level on naïve murine T cells and at a very low level on human T cells^9^, whereas a GITR ligand (GITRL) was abundantly expressed in murine dendritic cells and macrophages^10^. Multiple studies have shown that GITR-GITRL interaction can provide a co-stimulatory signal to both CD4^+^ and CD8^+^ naïve T cells, enhancing proliferation and effector function, particularly in the setting of suboptimal T cell receptor (TCR) stimulation^10^. In addition, GITR^−/−^ T cells are more prone to activation-induced cell death (AICD), suggesting that GITR signaling may protect T cells from AICD^10^. In contrast, murine and human Tregs constitutively express GITR, and it had been shown that activation of GITR signaling by GITR ligand or agonistic antibody inhibit the suppressive activity of Tregs^9^. Therefore, the induction of tumor immunity by GITR Ab is attributable to both the co-stimulatory activity of GITR on responder CD4^+^CD25^−^ T cells and to a direct effect on CD4^+^CD25^+^ Tregs^11–13^.

To enhance the antitumor effect of immune stimulatory reagents, we have been focusing on the intratumoral administration route^14^. Since the GITR agonistic Ab directly activates effector T cells and suppresses Tregs, the increase of Ab concentration in tumors and surrounding tissues including lymph nodes by the intratumoral route may enhance only the tumor-infiltrating T cells and break the tumor-specific immune-tolerant microenvironment. In this study, we compared intratumoral injection of anti-GITR agonistic antibody (local administration) with intraperitoneal and intravenous injection (systemic administration), and showed that the intratumoral route of anti-GITR agonistic antibody induced a more effective antitumor immunity than the systemic route did.

## Results

### Intratumoral injection of DTA-1 antibody more effectively suppressed tumor growth than did intraperitoneal injection

**Figure 1:**
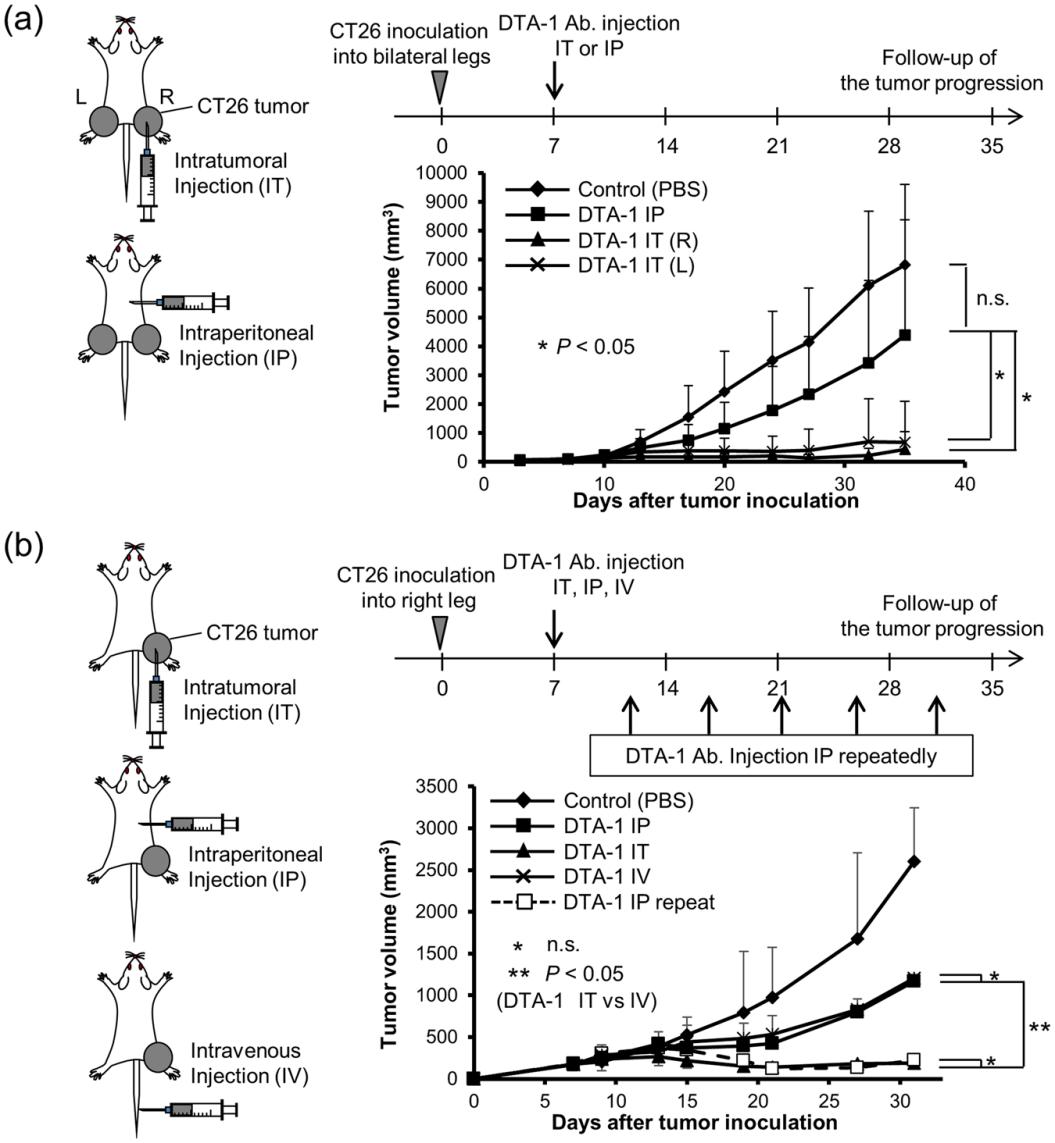
DTA-1 Ab enhanced antitumor immunity. (**a**) Antitumor effect of DTA-1 Ab. CT26 subcutaneous tumors were established on both legs of BALB/c mice. DTA-1 Ab was once injected into the right tumor (IT; intratumoral injection) or into the peritoneal cavity (IP; intraperitoneal injection). Tumor volumes were measured at the indicated days. n.s.: not significant. Control: n = 14, IP: n = 14, IT (R): n = 7, IT (L): n = 7. *P* < 0.05; one-sided ANOVA with Turkey’s post-hoc test. (**b**) Antitumor effect of repeated injections of DTA-1 Ab. CT26 subcutaneous tumors were established on right leg of BALB/c mice. DTA-1 Ab was once injected into the tumor, into the peritoneal cavity or into the tail vein (IV; intravenous injection), and DTA-1 Ab was repeatedly injected into the peritoneal cavity every 4–5 days (IP repeat). Tumor volumes were measured at the indicated days. n = 7. *P* < 0.05; one-sided ANOVA with Turkey’s post-hoc test.

First, to compare the difference of systemic antitumor effect by administration route, we subcutaneously inoculated CT26 cells on the bilateral legs, and injected 50 μg of DTA-1 Ab into the CT26 tumor on their right legs (local administration) or into their peritoneal cavity (systemic administration). Intraperitoneal injection of DTA-1 Ab slightly suppressed tumor growth, whereas intratumoral injection of DTA-1 Ab markedly suppressed the growth of not only DTA-1 Ab-injected tumors but also opposite Ab-uninjected tumors as an abscopal effect (Fig. 1a). Then, intravenous injection of DTA-1 Ab was compared with the intratumoral and intraperitoneal routes. The antitumor effect of intravenous injection was compatible with that of intraperitoneal injection (Fig. 1b). The results confirmed that local administration of DTA-1 Ab was more effective than systemic administration. Then, to examine whether the duration of DTA-1 Ab treatment influence the antitumor effect, we injection intraperitoneally 50 μg DTA-1 Ab every 4–5 days. The antitumor effect of repeated intraperitoneal injections showed a strong antitumor effect, which was compatible with that of single intratumoral injection (Fig. 1b), suggesting that the long term elevation of DTA-1 Ab concentration is associated with an induction of antitumor immunity.

### Intratumoral injection of DTA-1 antibody induced tumor-specific immunity

**Figure 2:**
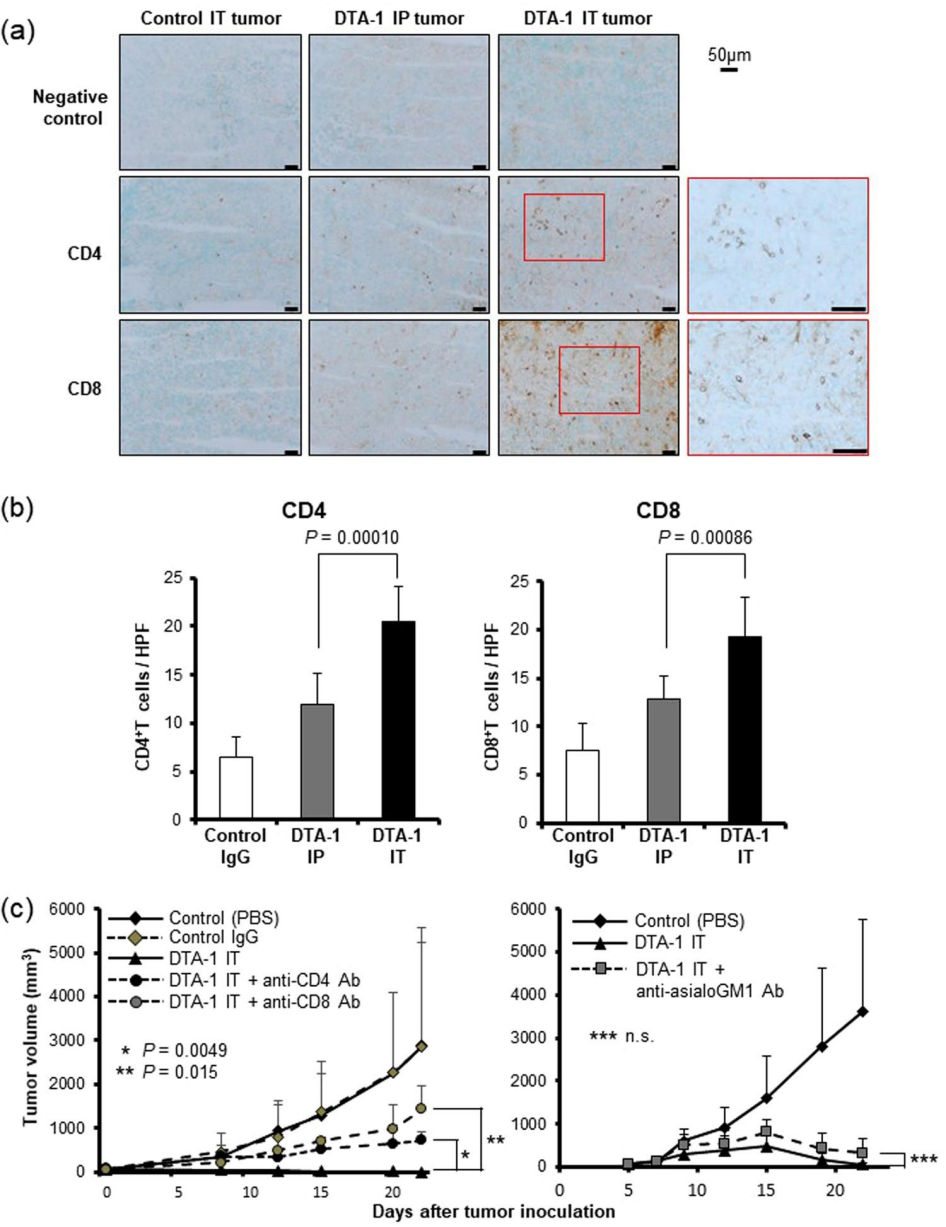
Intratumoral injection of DTA-1 Ab increased T cell-infiltration into tumors. (**a**) Immnohistochemical staining of CT26 tumors. Seven days after the administration of DTA-1 Ab, the fresh frozen sections of subcutaneous tumors were processed for immunohistochemistry with anti-CD4 and anti-CD8 antibodies. (**b**) Number of CD4^+^ and CD8^+^ T cells in CT26 tumors. Positive cells were counted in 8 representative high power view fields (HPF, x 400) under microscope. *P* values for DTA-1 IP vs IT, as analysed by *t*-test. (**c**) Antitumor effect of intratumoral DTA-1 Ab injection after *in vivo* depletion of effector cells. Left panel: the depletion of CD4^+^ or CD8^+^ T cells (n = 7). Right panel: the depletion of NK cells (n = 7). *P* values for DTA-1 IT vs DTA-1 IT + anti-CD4 Ab (*), DTA-1 IT vs DTA-1 IT + anti-CD8 Ab (**) and DTA-1 IT vs DTA-1 IT + anti-asialoGM1 Ab (***), as analysed by *t*-test.

To confirm that the antitumor effect of intratumoral injection was due to the induction of antitumor immune reaction, we performed the immunostaining of effector T cells in the treated tumors. The numbers of CD4^+^ and CD8^+^ T cells infiltrated in intratumorally DTA-1 Ab-injected tumors were significantly higher than those in the intraperitoneally DTA-1 Ab-administrated mice (Fig. 2a,b). Depletion of each CD4^+^ T cell or CD8^+^ T cell showed growth advantages and, in particular, CD8^+^ T cells appeared to contribute in antitumor immunity more strongly than CD4^+^ T cells (Fig. 2c left panel). The control rat IgG did not result in any antitumor effect compared with PBS. The growth of CT26 tumors in NK cell-depleted mice was markedly suppressed by the intratumoral DAT-1 Ab injection (Fig. 2c right panel), indicating that NK cells did not contribute to the antitumor effect of DTA-1 Ab. To evaluate the change of Tregs in tumors, we performed an immunostaining of Tregs in tumors treated with intratumoral and intraperitoneal injection of DTA-1 Ab. The intraperitoneal injection significantly decreased the number of Tregs, while intratumoral injection more reduced the number in tumors (Supplemental Fig. S1). The intratumoral injection of DTA-1 Ab seems to enhance the activation of effector cells, and suppress the infiltration of Tregs into tumors.

**Figure 3:**
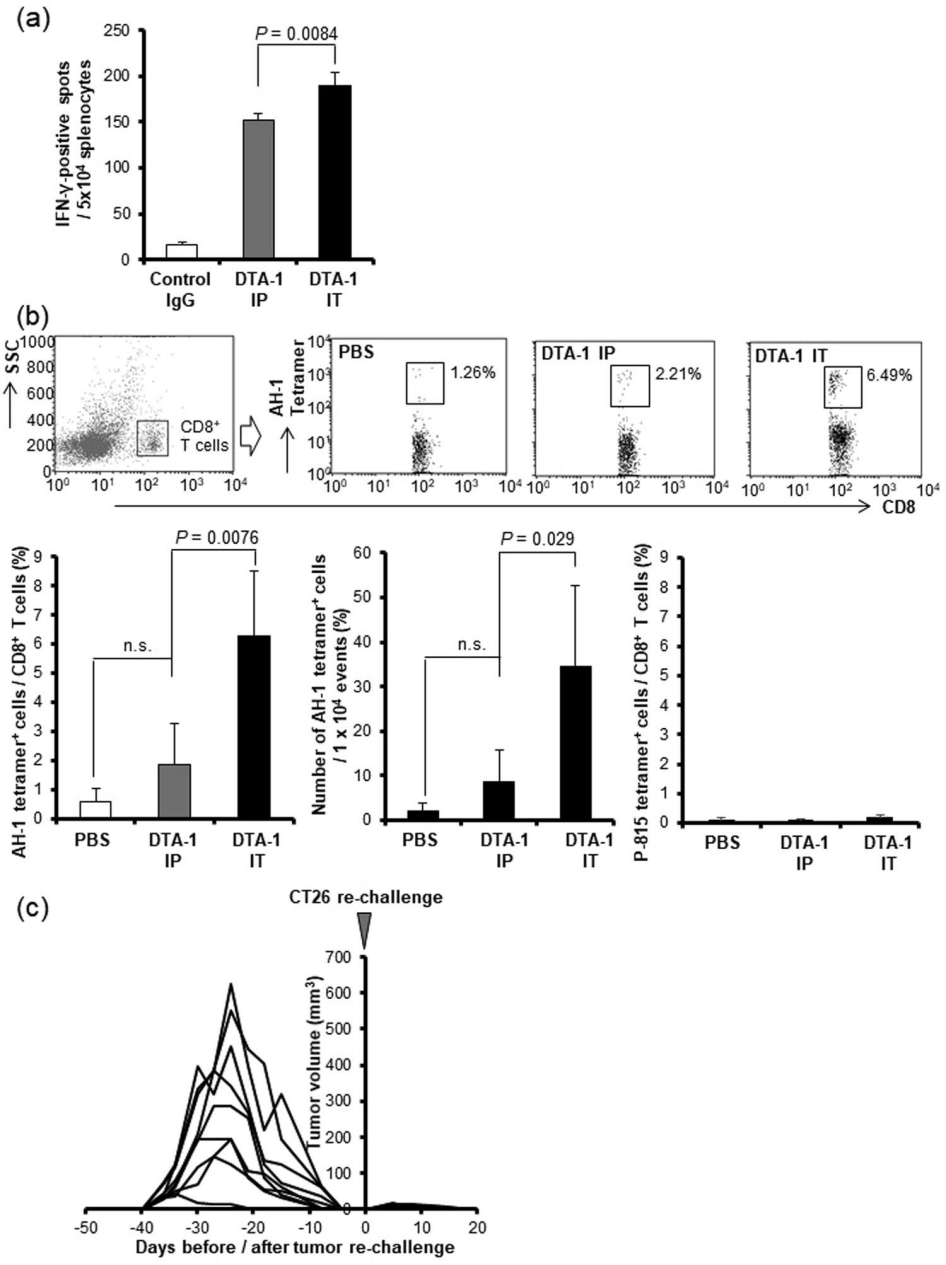
Intratumoral injection of DTA-1 Ab more effectively activated tumor-specific immunity. (**a**) IFN-γ ELISpot assay in response to stimulation of CT26 tumor cells. DTA-1 Ab was injected into the CT26 subcutaneous tumor (IT) or into the peritoneal cavity (IP). Seven days later splenocytes were co-cultured with CT26 tumor cells, and stained with biotinylated anti-mouse IFN-γ antibody to detect captured IFN-γ (n = 3). *P* value for DTA-1 IP vs IT, as analysed by *t*-test. (**b**) The increase of tumor-specific CD8^+^ T cells in DTA-1 Ab IT-treated mice. The splenocytes were isolated seven days after DTA-1 Ab administration, and CT26-specific AH-1-tetramer-positive cells were analyzed by flow cytometry (upper panel). The ratio of AH-1 tetramer^+^ cells among CD8^+^ cells (n = 4) (lower left panel). Total cell number of AH-1 tetramer^+^ CD8^+^ cells in 1 × 10^4^ events (n = 4) (lower middle panel). The ratio of p815 tetramer^+^ cells among CD8^+^ cells (n = 4) (lower right panel). *P* values for PBS vs DTA-1 IP and DTA-1 IP vs IT, as analysed by *t*-test. (**c**) Re-challenge of CT26 tumor cells into the mice cured by the DTA-1 Ab treatment. CT26 cells were re-challenged at a few days after tumor rejection (n = 9).

IFN-γ-ELISpot assay showed that intratumoral injection of DTA-1 Ab induced a higher number of CT26-responsive IFN-γ^+^ T cells (Fig. 3a). The frequency of AH-1 tetramer^+^ CD8^+^ T cells, which are CT26-specific T cells^15^, among CD8^+^ T cells were found more plentifully in the intratumoral DTA-1-treated mice as compared with the mice that received intrapertioneal administration (Fig. 3b upper and lower left panels). The total cell number of AH-1^+^CD8^+^ T cells per 1 × 10^4^ events in intratumorally injected mice was also significantly higher than that in intrapertioneally injected mice (Fig. 3b upper and lower middle panels). As a non-specific tetramer staining, a P815 tetramer assay, which detects the antigen-specific T cells for p815 mastocytoma derived from DBA-2 mice, was performed. The assay showed that DTA-1 Ab treatment did not induce immune reaction for P815 (Fig. 3b lower right panel).

To clarify that GITR Ab-induced antitumor immunity could be promising against the tumor recurrence, CT26 cells were re-challenged into the mice cured by the DTA-1 Ab treatment. All the mice, in which the CT26 tumor disappeared by the DTA-1 Ab treatment, rejected the re-challenge of CT26 tumor cells, suggesting that an immunological memory specific for the CT26 tumor was successfully induced by DTA-1 Ab treatment (Fig. 3c). These findings indicated that the intratumoral administration of DTA-1 Ab induced CT26-specific cellular immunity more effectively than the systemic administration did.

### The transfer of DTA-1 antibody into tumor-draining lymph nodes is essential for inducing a strong antitumor immunity

**Figure 4:**
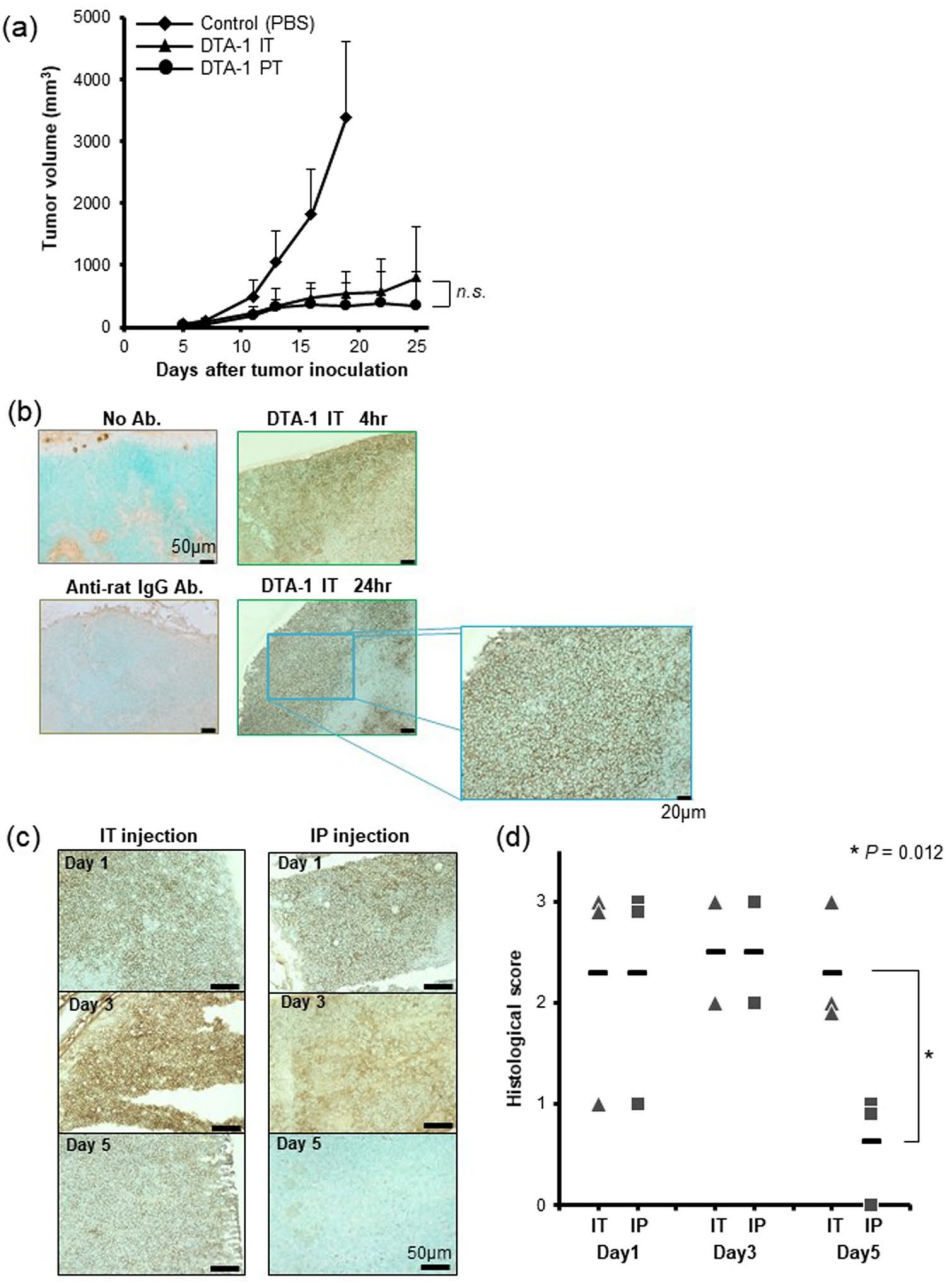
Intratumorally administrated DTA-1 Ab transferred into tumor draining lymph node. (**a**) Peritumoral injection of DTA-1 Ab. Fifty μg of DTA-1 Ab was injected directly into tumor (IT) or injected into subcutaneous space around the tumor (PT) (n = 8). Tumor volumes were measured at the indicated days. *P* value for DTA-1 IP vs PT, as analysed by *t*-test. (**b**) Immunohistochemical staining of tumor draining lymph nodes (TDLNs) for Rat IgG (DTA-1). DTA-1 Ab was intratumorally injected, and TDLNs were harvested 4 and 24 hours after Ab injection. (**c**) Detection of DTA-1 Ab in TDLNs. The transfer of DTA-1 Ab into TDLNs was compared between intraperitoneal injection (IP) and intratumoral injection (IT). Immunohistochemical staining of TDLNs was performed sequentially 1, 3 and 5 days after DTA-1 Ab injection. (**d**) Immunohistological score of DTA-1 Ab in TDLNs. Immunohistochemical scoring criteria are defined in Materials and Methods. Crossbar means the average. *P* value for DTA-1 IP vs IT on day 5, as analysed by *t*-test.

Next, to examine whether it is necessary for the administrated antibody to exist in the tumors for the induction of antitumor immunity, we compared the intratumoral injection with the peritumoral injection of DTA-1 Ab, and found that the peritumoral injection also significantly suppressed CT26 tumor growth, and was compatible with that of intratumoral injection (Fig. 4a). Therefore, we hypothesized that the transfer of locally administrated DTA-1 Ab into TDLNs is important to activate cellular immunity. Immunostaining detected intratumorally injected DTA-1 Ab (Rat IgG) in the cortex of TDLNs at an early phase (4 hours and 24 hours) of the treatment (Fig. 4b), and was also detected on day 5 after the injection, whereas intraperitoneally injected Ab was detected in the TDLNs on day 1 but the amount of Ab was much decreased on day 5 (Fig. 4c,d), indicating that the intratumoral delivery sustained a high concentration of DTA-1 Ab in TDLNs. The anti-rat IgG Ab alone did not show any non-specific staining. Peritumorally injected DTA-1 Ab was also detected in the TDLNs even on day 5 after the injection, which was similar with the intratumoral injection (Supplemental Fig. S2). Regarding with tumor, a marked staining of DTA-1 was recognized in tumors treated with intratumoral injection 5 days after Ab injection, whereas only faint staining was detected in intraperitoneally injected tumors (data not shown). The results suggested that locally administrated Ab within and around tumor region may release gradually the Ab from inner part of the tumor or peritumoral tissue into TDLNs for more than 5 days.

**Figure 5:**
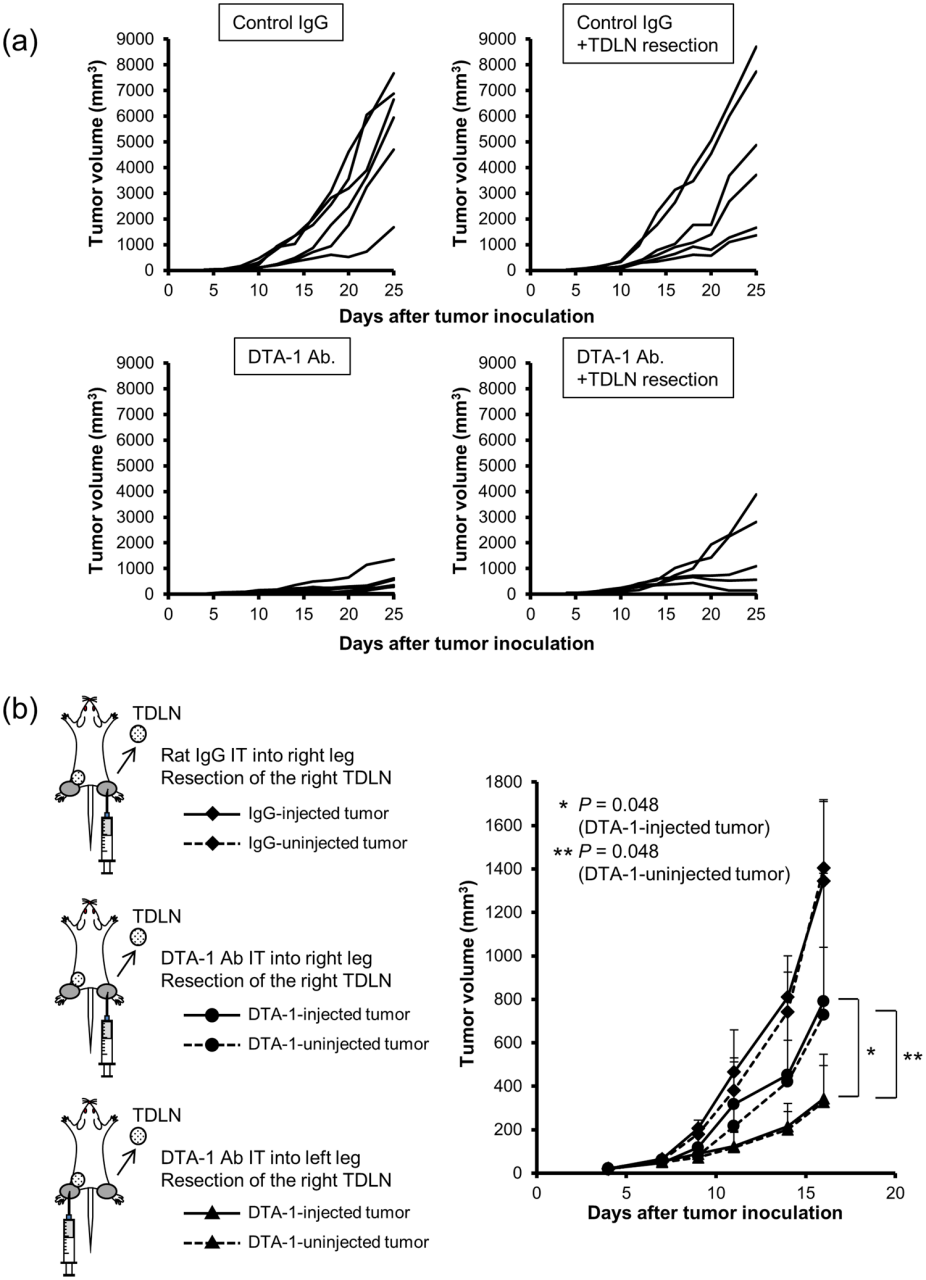
Resection of DTA-1-containing TDLN reduced antitumor immunity. (**a**) The attenuation of antitumor effect of DTA-1-treated TDLN resection. DTA-1 Ab was injected into tumors 7 days after tumor inoculation, following TDLN resection 4 hour after Ab injection (n = 6). Tumor volumes were measured at the indicated days. (**b**) The effect of DTA-1 Ab-injected TDLNs on antitumor effect. CT26 subcutaneous tumors were established on both legs of BALB/c mice. Then, DTA-1 Ab was injected into one side, following TDLN resection of the same side (black circle) or the opposite side (black triangle) (n = 6). *P* values for volume of DTA-1-injected tumor with removal of TDLNs of injected tumors vs DTA-1-injected tumor with removal of TDLNs of uninjected tumors (*), and volume of DTA-1-uninjected tumor with removal of TDLNs of injected tumors vs DTA-1-uninjected tumor with removal of TDLNs of uninjected tumors (**), as analysed by *t*-test.

To confirm that the transfer of DTA-1 Ab into TDLNs is essential for inducing antitumor immunity, TDLN was resected immediately after the intratumoral injection of Ab. Resection of control IgG-injected TDLNs did not change the tumor growth (Fig. 5a upper left and upper right panels), suggesting that the TDLNs induce no significant antitumor immunity in control mice. In contrast, although not significant on day 25, the average of tumor volume between DTA-1 Ab and DTA-1 Ab + TDLN resection groups was statistically significant on day 16 (*P* = 0.049) and on day 18 (*P* = 0.039) (Fig. 5a lower left and lower right panels), suggesting that resection of DTA-1 Ab-injected TDLN reduced the Ab-mediated antitumor effect. Then, we inoculated CT26 tumor cells into both legs of BALB/c mice, injected DTA-1 Ab into one side of the tumors, and resected TDLNs on the same or opposite side of the DTA-1-injected tumor 4 hours after Ab injection. Resection of TDLNs on the side of Ab-uninjected tumors did not affect the antitumor effect induced by intratumorally administrated DTA-1. On the other hand, resection of TDLNs on the side of Ab-injected tumors significantly decreased their antitumor effect (Fig. 5b). These findings indicated that intratumorally delivered DTA-1 Ab transferred to TDLNs and induced a tumor immunity.

### Intratumoral injection of DTA-1 Ab showed less of an autoimmune reaction

**Figure 6:**
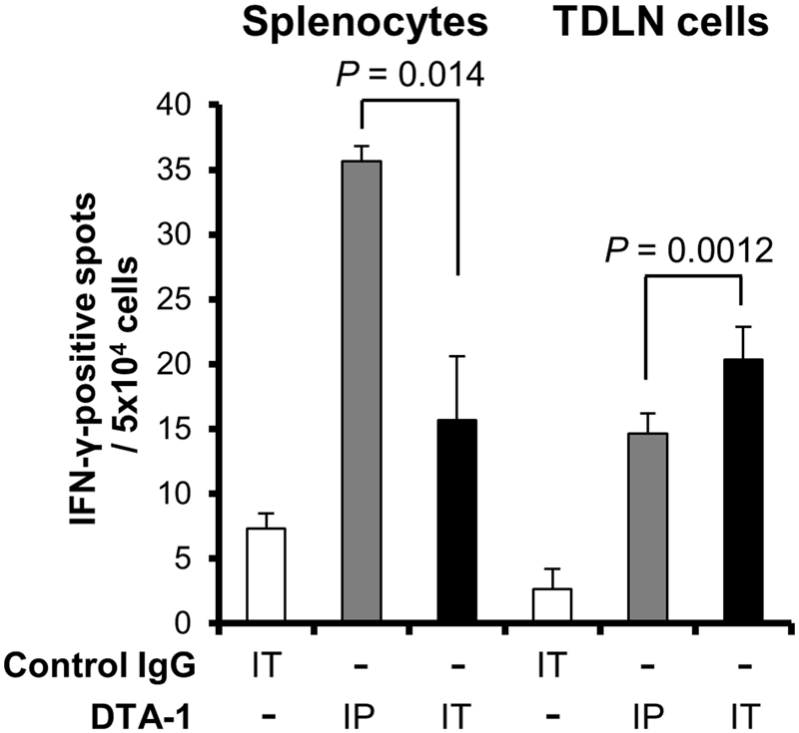
Less autoimmune reaction of intratumoral injection route. Auto-reactive T cell activation by the administration of DTA-1 Ab. DTA-1 Ab was injected into the CT26 subcutaneous tumor (IT) or into the peritoneum (IP), and 7 days later splenocytes and lymphocytes from TDLNs were co-cultured with syngeneic splenocytes. Nonspecific T cell activation was detected by IFN-γ-ELISpot assay (n = 3). *P* values for DTA-1 IP vs IT, as analysed by *t*-test.

The GITR agonistic antibody may cause autoimmune reactions, due to the suppression of Treg activity. In fact, in previous reports, high dose of GITR agonistic antibody resulted in autoimmune gastritis and anaphylaxis in young mice^12,16^. To evaluate the autoimmune response by DTA-1 Ab, splenocytes and TDLN cells were collected from DTA-1-treated mice, and T cell activation against syngeneic lymphocytes was analyzed. ELISpot assay showed that in TDLNs, auto-reactive T cells were detected in mice treated with intratumoral and intraperitoneal injection of DTA-1, but the difference of the IFN-γ^+^ cell number was minimal (Fig. 6). On the other hand, the higher number of auto-reactive T cells was detected in the spleen of mice that received the intraperitoneal injection of DTA-1 Ab as compared with the intratumoral injection (Fig. 6).

All treated mice looked healthy during the course of the experiments, and showed no abnormal physical findings including diarrhea and bloody stool. Hematoxylin-eosin staining of the stomachs and lungs revealed no significant findings such as the infiltration of immune cells in intratumorally and intraperitoneally Ab-injected mice (data not shown). Although Shimizu *et al*. reported that administration of DTA-1 Ab (1 mg per inoculation) to young BALB/c mice for once a week for 3 weeks from 2 weeks of age induced histological evidence of autoimmune gastritis^12^, in this study we used a lower dose (50 μg) of Ab in 5 to 6-week-old mice, which may be a reason behind a lack of autoimmune lesions. Since the intratumoral injection of GITR Ab induced a strong antitumor immunity, in the clinical setting the intratumoral route can reduce the dose of GITR Ab, which may enhance patient security.

## Discussion

Intratumoral delivery route has been investigated as a strategy to enhance antitumor immunity. In the intratumoral immunotherapy, the cancer vaccine is generated *in vivo* without the need to previously identify and isolate tumpr-associated antigens (TAAs). The release of abundant TAAs in the tumor site from the intratumorral immunotherapy can enhance antigen uptake by antigen presenting cells and effectively induce a systemic response. This strategy can take advantage of the complete antigenic repertoire of a tumor and not be limited to a single TAA. Intratumoral immunotherapy can be carried out while working with much less dose than systemic dose, which may reduce a risk of immune-related adverse effects^17,18^. Recently, considering these advantages, intratumoral immunotherapy using tumor lysate-pulsed DCs, cytokines, TLRs, oncolytic viruses has been under study^17,18^. Furthermore, Tuve *et al*. showed for the first time that expression of a CTLA-4 antibody localized to a tumor site exerts effective antitumor immune responses in mice^19^. Marabelle *et al*. also reported that intratumoral co-injection of anti-CTLA-4 and anti-OX40 Ab together with CpG depleted tumor-infiltrating Tregs and concluded that immunomodulating Ab could be used to target the tumor infiltrative immune cells locally, thereby eliciting a systemic immune response^20^. In this study also, intratumoral injection of DTA-1 Ab significantly induced a stronger antitumor immunity (Fig. 1c). Since the GITR agonistic antibody can strongly activate effector T-cells, the combination of immune checkpoint inhibitors and GITR agonistic Ab may be promising. In the next preclinical study, we are going to employ the more clinically relevant models with the target cancer type and animal models resembling clinical situations.

Why was the intratumoral delivery of anti-GITR agonistic antibody more able to effectively induce an antitumor immunity than the systemic delivery? We focused on the place where the administrated anti-GITR agonistic antibody would act *in vivo*. Although it is not fully understood where the immune modulatory reagents exert the function, intratumoral and peritumoral injections of DTA-1 Ab showed a strong antitumor effect in this study, suggesting that the anti-GITR agonistic antibody worked inside tumors and TDLNs. In fact, immunostaining demonstrated that DTA-1 Ab was detected in TDLNs for more than 5 days after intratumoral injection (Fig. 4c). Furthermore, the resection of DTA-1 Ab-injected TDLNs reduced the antitumor effect at both sites of the tumors, whereas the resection of Ab-uninjected TDLNs did not affect the antitumor effect (Fig. 5a,b). These results suggested that TDLNs of the Ab-injected tumors play a significant role in inducing an effective antitumor immunity. Resection of TDLNs on DTA-1 Ab-injected tumors significantly but partially decreased their antitumor effect, namely TDLN resection could not completely cancel the antitumor effect of DTA-1 injection. One of the reasons was thought that locally injected Ab was mainly distributed TDLNs, but in some extent, the DTA-1 Ab may distribute to other LNs or systemic circulation, in where the Ab stimulates tumor-reactive T cells.

It is well-known that dendritic cells (DCs) migrate from tumors into TDLNs and present TAAs to T cells there^21^. To confirm whether DTA-1 Ab does not influence the activation status of DCs, we examined the change of cytokine production from DCs *in vitro*. The production of cytokines (IFN-γ, IL-12p70, IL-1β, IL-6, KC, TNF-α) from DCs was not changed by DTA-1 Ab (MSD SECTOR Imager 2400 instrument, Meso Scale Discovery, Inc.) (our data not shown and ref.^22^), which suggested that GITR Ab did not directly affect the function of DCs. However, since GITR Ab may influence the function of DCs via interactions with cytokines and other immune cells in the tumor microenvironment, it is necessary to investigate further the effect of GITR Ab on DCs in tumor sites.

Regarding another mechanism of effective antitumor immunity induction by intratumoral delivery of DTA-1 Ab, GITR-GITRL interaction on endothelial cells is important for triggering leukocyte adhesion and transmigration^23^. It is known that a large number of tumor-infiltrating lymphocytes (TILs) results in a better prognosis for cancer patients^24^, and that patients with a high number of effector cells in tumors were more likely to respond favorably to anti-CTLA4 Ab^25^. GITR stimulation on endothelial cells in a tumor and TDLNs by DTA-1 Ab may promote the migration there of effector cells from the blood circulation.

Contrary to the clinical effectiveness of anti-CTLA-4 Ab, several immunotoxicities such as autoimmune colitis, dermatitis, hepatitis, hypophysitis and thyrotoxicosis were observed in the treated patients^26–29^. In particular, anti-CTLA-4 Ab-induced hypophysitis is a common serious side effect with frequent hormonal deficiencies, and the risk of adrenal insufficiency remains in the long-term^30^. Therefore, it is necessary to develop a feasible approach for enhancing the antitumor immunity of immune modulatory therapy without the exacerbation of immunotoxicities. Regarding with auto-reactive T cells, the number of IFN-γ^+^ spots detected in TDLNs of intratumorally Ab-treated mice was slightly larger than that of intraperitoneally Ab-treated mice (Fig. 6). This is probably due to the high concentration of DTA-1 antibody in TDLNs of intratumorally Ab-treated mice compared with intraperitoneally Ab-treated mice, which may induce the activation and proliferation of tumor-specific T cells as well as auto-reactive T cells. In the spleen, intraperitoneal injection of DTA-1 Ab increased the number of auto-reactive T cells than intratumoral injection did. This discrepancy between the TDLN and spleen may be explained by the higher concentration of DTA-1 Ab in the systemic circulation in intraperitoneally Ab-treated mice, inducing the activation of auto-reactive T cells in a variety of lymphoid organs.

Since the intratumoral injection of DTA-1 Ab induced much stronger antitumor immunity than the systemic administration did, intratumoral route may be able to decrease the administration dose of DTA-1 Ab to achieve the same antitumor efficacy as the systemic injection did, which may reduce the risk of occurrence of autoimmune reaction in the clinical setting. As a next step, we are going to assess the superiority of intratumoral route compared with systemic administration from viewpoint of adverse effect using transgenic mice models on autoimmunity.

In conclusion, intratumoral delivery of GITR agonistic antibody induced a strong antitumor immunity via a transfer into TDLNs. Our preclinical study suggests that the intratumoral route of immune-modulating reagents is one of the promising approaches for the development of cancer immunotherapy.

## Materials and Methods

### Animals and a tumor cell line

Five-to-six-week-old female BALB/c mice were purchased from Charles River Japan, Inc., (Kanagawa, Japan). Animal studies were carried out according to the Guideline for Animal Experiments of the National Cancer Center Research Institute and approved by the Institutional Committee for Ethics in Animal Experimentation at National Cancer Center Research Institute (Tokyo, Japan).

CT26 is a weakly immunogenic BALB/c-derived colon cancer cell line, which was obtained from the American Type Culture Collection (Rockville, MD, USA). Cells were maintained in RPMI containing 10% fetal bovine serum, 2 mM L-glutamine and 0.15% sodium bicarbonate (complete RPMI).

### Tumor inoculation and administration of anti-GITR agonistic antibody

CT26 cells (1 × 10^6^) were injected subcutaneously into the leg(s) of BALB/c mice. Seven days after tumor inoculation, tumor size when reached 50 mm^3^, the mice received intraperitoneal, intravenous, intratumoral, or peritumoral injection of monoclonal antibody from DTA-1 hybridoma. DTA-1 Ab (rat IgG) was prepared by the 50 μg of DTA-1 Ab into a total of 200 μl (intraperitoneal and intravenous injection) or 50 μl (intratumoral and peritumoral injection) of phosphate-buffered saline per mouse and injected by 27G (intraperitoneal and intravenous injection) or 29G (intratumoral and peritumoral injection) needles. The shortest (*r*) and longest (*l*) tumor diameters were measured at indicated days and the tumor volume was determined as *r*^2^*l*/2. Data are presented as mean ± s.d.

### *In vivo* depletion of T cells and NK cells

To identify the effector cells, which directly attack tumor cells, we depleted the subsets of immune effector cells during the treatment with GITR Ab. The mice received intraperitoneal injections of 0.3 mg monoclonal antibody (mAb) from the anti-CD4^+^ hybridoma (clone GK1.5, rat IgG2b), 1.5 mg mAb from the anti-CD8^+^ hybridoma (clone Lyt-2.1, mouse IgG2b) or 0.5 mg of anti-asialo GM1 antibody (targeting NK cells: Wako Pure Chemical Industries, Ltd., Tokyo, Japan). Administration of mAbs started at 5 days after the inoculation of CT26 cells, and the injection was repeated every 5–6 days, throughout the entire experimental period. Flow cytometry showed that ~80% of CD4^+^, ~60% of CD8^+^ T cells and ~80% of NK cells were depleted in the spleen of Ab-treated mice.

### Enzyme-linked immunospot assay (ELISpot assay)

IFN-γ ELISpot kit (BD Bioscience) was used according to the manufacturer’s instructions. Briefly, splenocytes or lymphocytes derived from tumor-draining lymph nodes (1 × 10^5^) and mitomycin C-treated tumor cells (1 × 10^4^) were co-cultured in 96-well plates pre-coated with mouse anti-IFN-γ antibody (BD Bioscience) for 20 h at 37 °C in complete RPMI medium in triplicate. After washing the wells, biotinylated anti-mouse IFN-γ antibody (2 μg/ml) was added and incubated for 2 h at room temperature. Then, a streptavidin-horseradish peroxidase solution was added and incubated for 1 h at room temperature. After the addition of an aminoethly carbazole substrate solution, spots were counted under a stereomicroscope.

### Tetramer staining and fluorescence-activated cell sorting analysis

The CT26-specific H-2Ld MuLVgp70 (AH-1) peptide tetramer and H-2Ld p815 peptide tetramer were purchased from MBL Inc. (Nagoya, Japan), and fluorescein isothiocyanate (FITC)-anti mouse CD8 antibody were from BD Biosciences. For cell staining, the manufacturer’s protocol was followed. Spleens were harvested from the mice, and after washing, the cells were incubated with tetramer and antibody in a total volume of 100 μl PBS with 2% FBS for 30 min at 4 °C and then fixed. Cells were analyzed by fluorescence-activated cell sorting (FACSCalibur; BD Bioscience). Irrelevant IgG was used as a negative control. Ten thousand live events were acquired for analysis. The ratio of tetramer^+^ cells was calculated as the frequency of AH-1^+^ or P815^+^ cells among CD8^+^ T cells.

### Immunohistochemistry and the assessment of transferred DTA-1 antibody into lymph nodes

Immunostaining was performed using the streptavidin-biotin-peroxidase complex techniques (Nichirei, Tokyo, Japan). Consecutive cryostat tissue sections (5 μm) were mounted on glass slides and fixed in 99.5% ethanol for 20 min. After blocking with normal rat serum, the sections were stained with rat anti-mouse CD4 and CD8 (BD Biosciences). Negative controls without primary antibodies were examined in all cases. The sections were counterstained with methylgreen.

Immunohistochemical staining of tumor-draining lymph nodes (TDLNs) for Rat IgG (DTA-1) was performed using the streptavidin-biotin-peroxidase complex techniques (Nichirei). Immunohistochemical scoring criteria defined as the following, 3+: Circumferential membrane staining that is complete, intense, and within >10% of lymph node cells. 2+: Circumferential membrane staining that is incomplete and/or weak/moderate and within >10% of lymph node cells, or complete and circumferential membrane staining that is intense, and within ≦10% of lymph node cells. 1+: Incomplete membrane staining that is faint/barely perceptible and within >10% of lymph node cells. 0: No staining is observed, or membrane staining that is incomplete and is faint/barely perceptible and within ≦10% of lymph node cells. The histologic scoring was evaluated by a pathologist (N. H.) in a blinded manner.

### Statistical analysis

Comparative analyses of the data were performed by one-way ANOVA with Tukey’s post-hoc test for multiple pairwise testing, and the Student’s *t*-test when two groups were compared, using SPSS statistical software (SPSS Japan Inc., Tokyo, Japan). P < 0.05 was considered as a significant difference.

## Supplementary information

Supplementary Dataset 1
