# Supplementary material for: Local Administration of GITR Agonistic Antibody Induces a Stronger Antitumor Immunity than Systemic Delivery
Source: Sci Rep. 2019 Apr 3;9:5562. doi: 10.1038/s41598-019-41724-x (PMC6447616; doi:10.1038/s41598-019-41724-x)
Supplement: Supplementary file 1 — Supplementary Dataset 1 [file 41598_2019_41724_MOESM1_ESM.pdf]

# Local Administration of GITR Agonistic Antibody Induces a Stronger Antitumor Immunity than Systemic Delivery

Kenta Narumi[1], Reina Miyakawa[1], Chihiro Shibasaki[1], Marina Henmi[1], Yukihiro Mizoguchi[1], Ryosuke Ueda[1], Hisayoshi Hashimoto[1], Nobuyoshi Hiraoka[2], Teruhiko Yoshida[3], Kazunori Aoki[1*]

[1]Division of Molecular and Cellular Medicine, [2]Division of Molecular Pathology, [3]Division of Genetics, National Cancer Center Research Institute, 5-1-1 Tsukiji, Chuo-ku, Tokyo 104-0045, Japan

Correspondence: Kazunori Aoki, Division of Molecular and Cellular Medicine, National Cancer Center Research Institute
5-1-1 Tsukiji, Chuo-ku, Tokyo 104-0045, Japan
Tel: +81-3-3542-2511, Fax: +81-3-3248-1648
E-mail: kaoki@ncc.go.jp

Supplemental figure S1

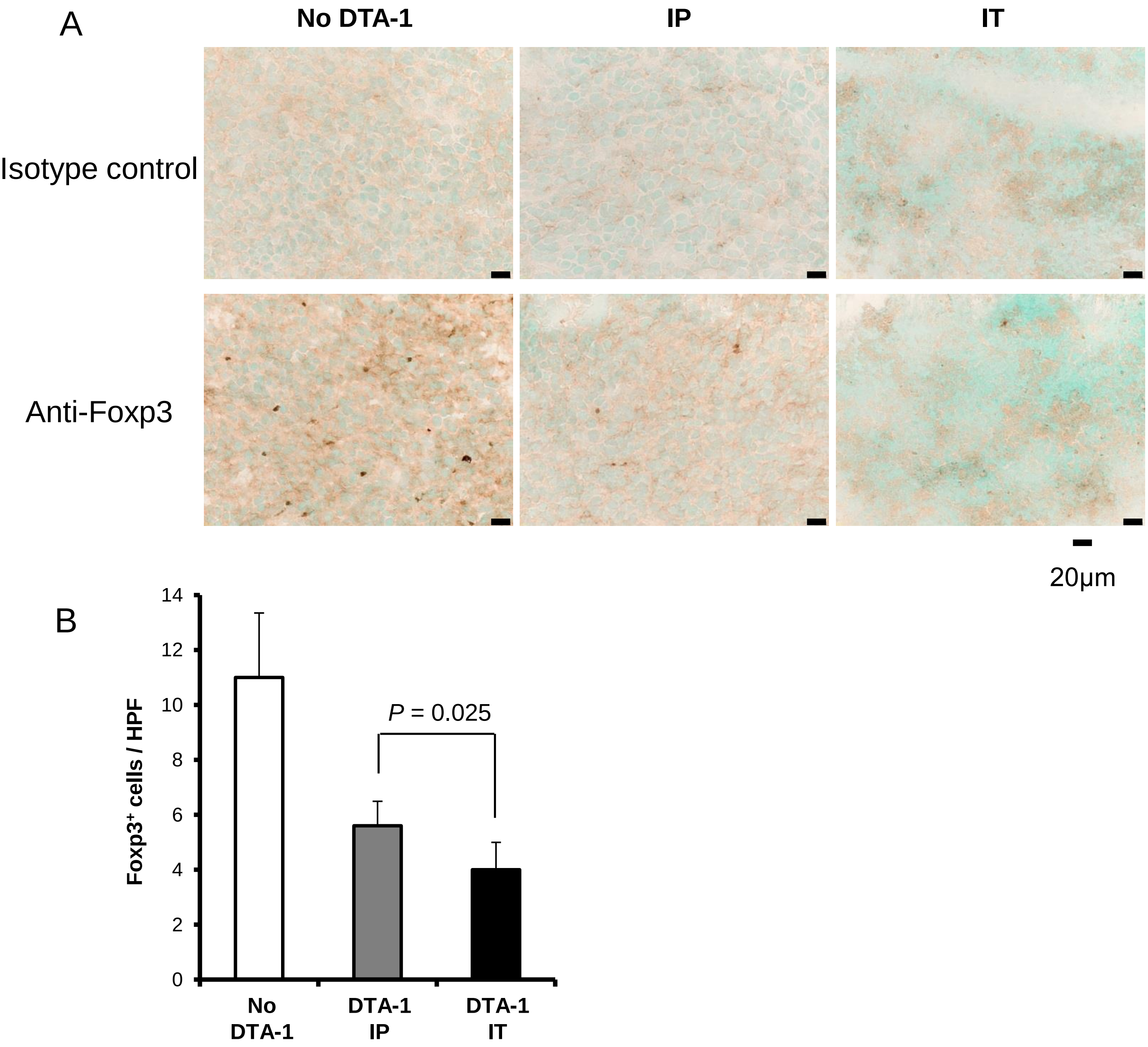


**Figure S1. Infiltration of Tregs into the DTA-1-treated tumors.**
(A) Tumors were harvested 5 days after Ab treatment, the fresh frozen sections of subcutaneous tumors were processed for immunohistochemistry with anti-Foxp3 antibody (BD Biosciences). Parallel negative controls with an antibody of the same isotype were examined in all cases. The sections were counter-stained with methylgreen. (B) Number of Foxp3+ cells in CT26 tumors. Positive cells were counted in 5 representative high power view fields under microscope.

# Supplemental figure S2

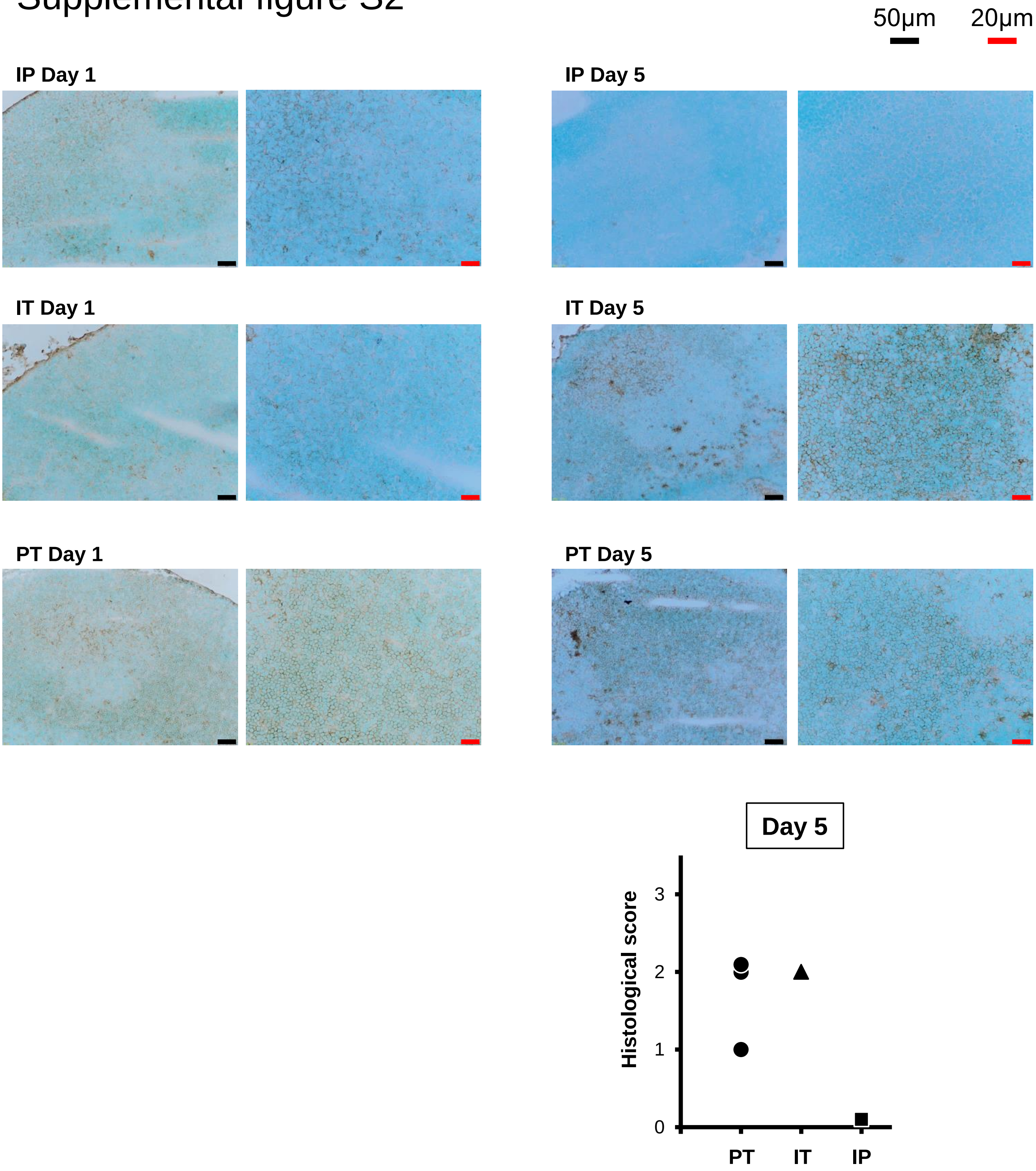


**Figure S2. Peritumorally injected DTA-1 Ab was also detected in the TDLNs on day 5 after the injection.**
Immunohistochemical staining of TDLNs for rat IgG (DTA-1) was performed sequentially 1, 3 and 5 days after DTA-1 Ab injection. Immunohistological scoring criteria are defined in Materials and Methods. The sections were counterstained with methylgreen.
